# Structured relearning of activities of daily living in dementia: the randomized controlled REDALI-DEM trial on errorless learning

**DOI:** 10.1186/s13195-017-0247-9

**Published:** 2017-03-23

**Authors:** Sebastian Voigt-Radloff, Maartje M. E. de Werd, Rainer Leonhart, Danielle H. E. Boelen, Marcel G. M. Olde Rikkert, Klaus Fliessbach, Stefan Klöppel, Bernhard Heimbach, Andreas Fellgiebel, Richard Dodel, Gerhard W. Eschweiler, Lucrezia Hausner, Roy P. C. Kessels, Michael Hüll

**Affiliations:** 1 Center for Geriatric Medicine and Gerontology, University Medical Center Freiburg, Freiburg, Germany; 2 Department of Medical Psychology & Radboudumc Alzheimer Center, Radboud University Medical Center, P.O. Box 9101 (internal post 925), 6500 HB Nijmegen, The Netherlands; 3 Department of Geriatric Medicine & Radboudumc Alzheimer Center, Radboud University Medical Center, Nijmegen, The Netherlands; 4 Department of Social Psychology & Methodology, University of Freiburg, Psychological Institute, Freiburg, Germany; 5 Rehabilitation Center Klimmendaal, Arnhem, The Netherlands; 6 Department of Psychiatry and Psychotherapy, University Medical Center Bonn, Bonn, Germany; 7 German Center for Neurodegenerative Diseases (DZNE), Bonn, Germany; 8 Department of Psychiatry and Psychotherapy, University Medical Centre Freiburg, Freiburg, Germany; 9 Department of Psychiatry and Psychotherapy, University Medical Center Mainz, Mainz, Germany; 10 Department of Neurology, Philipps University Marburg, Marburg, Germany; 11 Department of Geriatrics, University Clinic Essen, Essen, Germany; 12 Geriatric Center, University Hospital Tübingen, Tübingen, Germany; 13 Department of Gerontopsychiatry, Central Institute of Mental Health, Medical Faculty Mannheim, Heidelberg University, Mannheim, Germany; 14 Donders Institute for Brain, Cognition and Behaviour, Radboud University, Nijmegen, The Netherlands

**Keywords:** Alzheimer’s dementia, Errorless learning, Activities of daily living, Dementia, Randomized controlled trial, Cognitive rehabilitation

## Abstract

**Background:**

Errorless learning (EL) is a method for optimizing learning, which uses feed-forward instructions in order to prevent people from making mistakes during the learning process. The majority of previous studies on EL taught patients with dementia artificial tasks of little or no relevance for their daily lives. Furthermore, only a few controlled studies on EL have so far been performed and just a handful of studies have examined the long-term effects of EL. Tasks were not always trained in the patients’ natural or home environment, limiting the external validity of these studies. This multicenter parallel randomized controlled trial examines the effects of EL compared with trial and error learning (TEL) on the performance of activities of daily living in persons with Alzheimer’s or mixed-type dementia living at home.

**Methods:**

Patients received nine 1-hour task training sessions over eight weeks using EL or TEL. Task performance was measured using video observations at week 16. Secondary outcome measures were task performance measured at week 26, satisfaction with treatment, need for assistance, challenging behavior, adverse events, resource utilization and treatment costs.

**Results:**

A total of 161 participants were randomized, of whom 71 completed the EL and 74 the TEL arm at week 11. Sixty-nine EL patients and 71 TEL patients were assessed at the 16-week follow-up (the primary measurement endpoint). Intention-to-treat analysis showed a significantly improved task performance in both groups. No significant differences between the treatment groups were found for primary or secondary outcomes.

**Conclusions:**

Structured relearning improved the performance of activities of daily living. Improvements were maintained for 6 months. EL had no additional effect over TEL.

**Trial registration:**

German Register of Clinical Trials DRKS00003117. Registered 31 May 2011.

**Electronic supplementary material:**

The online version of this article (doi:10.1186/s13195-017-0247-9) contains supplementary material, which is available to authorized users.

## Background

The increasing deterioration of cognitive and daily functioning in Alzheimer’s dementia (AD) causes the main burden for patients, their caregivers and society, while options for disease-modifying treatments are still lacking [1, 2]. Evidence from systematic reviews of small-scale clinical trials suggests that structured teaching techniques may optimize or even stabilize daily functioning in AD [3–5]. Errorless learning (EL) is a prominent method for optimizing learning, which uses feed-forward instructions in order to prevent people from making mistakes during the learning process. It is assumed that by preventing errors during learning, the limited cognitive capacity of AD patients is directed toward the acquisition of the correct steps of a task, without interference of occurring errors [4]. The rationale behind EL is that explicit memory is responsible for recognizing and correcting the errors that are made during learning. In people with AD who have profound deficits in explicit memory, these errors may not be recognized as such and are therefore not corrected, but instead are implicitly consolidated into long-term memory. EL may include different techniques such as graded tasks broken down into small steps, modeling, encouragement not to guess, anticipating errors and immediate correction, prompts when steps are performed successfully, vanishing cues and spaced retrieval (rehearsal of the retrieval of information using increasing time intervals) [4, 5].

A meta-analysis on the treatment effects of EL and the method of vanishing cues in amnesic patients (*N* = 192) showed a large and beneficial effect for the EL treatment compared with trial and error learning (TEL) [3]. A qualitative review [5] included 26 studies on teaching persons with dementia daily tasks or skills. Five controlled group studies and 12 single-case studies obtained significantly superior effects of EL immediately after training compared with TEL or a no-treatment condition. Seventeen studies showed maintenance of EL effects at follow-up. Clare and Jones [4] performed a critical review including 15 empirical studies using group designs to compare the efficacy of EL and errorful learning in persons with brain injury or dementia. These authors argued that EL may be particularly beneficial in individuals with severe memory impairments. They concluded that benefits of EL for persons with early-stage and moderate dementia are mixed, with some studies finding an EL benefit and others reporting no additional advantage of EL.

The majority of previous studies taught dementia patients artificial tasks of little or no relevance for patients in daily life. Furthermore, only a few controlled studies on EL have so far been performed and just a handful of studies have examined the long-term effects of EL. Moreover, large differences were found across studies in the types of tasks that were taught and the exact errorless teaching methods that were used. Tasks were not always trained in the patients’ natural or home environment, limiting the external validity of these studies. Therefore we conducted the REDALI-DEM trial (RElearning methods on DAily LIving task performance of persons with DEMentia), a multisite randomized controlled trial (RCT) with the aim of comparing the effects of EL vs TEL on the performance of activities of daily living in persons with mild to moderate dementia living at home. Based on earlier studies [4, 5] we hypothesized that EL is superior compared with TEL.

Secondary questions of interest were as follows: can effects on performance be maintained for six months? Does relearning of daily living tasks show transfer effects to the patients’ initiative or need for assistance in activities of daily living? What are the treatment costs? How is the treatment accepted by patients? What adverse events occur during the treatment period?

## Methods

### Design

We used a six-center, single-blind, active-controlled design with a 1:1 randomization for two parallel groups to compare the effects of EL and TEL. The study was registered in the German Register of Clinical Trials (DRKS00003117), which is connected to the International Clinical Trials Registry Platform. The a-priori published study protocol is available elsewhere [6]. Prior to the RCT, a pilot study was performed in which the study procedure including the EL and TEL interventions was evaluated and monitored. The practical issues and difficulties that the therapists encountered were discussed, leading to minor protocol amendments after the 6-month trial pilot phase and before the start of recruitment; these concerned the participating study sites (two resigned due to organizational reasons, and to safeguard sufficient power a new site was included), inclusion criteria (the threshold for the need for assistance in activities of daily living was increased), intervention procedures (time to select training activities was extended from one to three sessions; the number of refresher sessions was reduced from three to two; special cue card series were not used in the EL arm) and outcome measurement (the task performance scale (TPS) was specified).

Previously available outcome measures to assess task performance have not been investigated with respect to reliability and construct validity in naturalistic settings using daily-life tasks. This pilot phase was therefore also used to validate the newly developed outcome measure: the Core Elements Method (CEM). The interrater reliability and concurrent validity of the CEM and TPS were analyzed and compared [7]. Based on these results, the TPS and CEM were found to be equally valid for the assessment of task performance in people with dementia. However, the CEM was found to be less complex and less time-consuming compared with the TPS, and was therefore used in the current RCT.

### Participants and setting

Persons living at home and diagnosed with mild to moderate AD or mixed-type dementia (Mini Mental State Examination (MMSE) [8] scores between 14 and 24) were eligible. Informed consent from both the patient and the primary caregiver was required. A caregiver had to be available for rating the need for assistance in activities of daily living. For inclusion, the mean score of the five household items in the performance scale of the Interview for Deterioration in Daily Living Activities in Dementia (IDDD) [9] had to be 2.5 or higher.

Exclusion criteria were major depression (Geriatric Depression Scale—Short Form (GDS-15) ≥ 9) [10], major need for physical nursing care (≥120 min per day) as well as severe behavioral disturbances, unstable medical conditions or lack of attention and understanding of instructions in German as judged by the recruiting study physician and involvement in other clinical trials.

The REDALI-DEM study sites were six outpatient memory centers at university hospitals; they are located throughout Germany in urban regions with catchment areas of about 100,000 (Marburg and Tübingen), 300,000 (Freiburg and Mainz) and 400,000 (Bonn and Mannheim) inhabitants, and all centers have provided outpatient dementia care for 5–17 years. The standard service of the study sites comprised a diagnostic work-up for dementia and related diagnoses as well as recommendations for risk reduction, dementia medication and nonpharmacological treatments. Principal investigators from the centers were psychiatrists, neurologists or geriatricians with long-standing experience in dementia care.

### Procedures

After patient recruitment, the site investigator requested randomization via email. Within 48 hours, the trial statistician at a detached site provided a 1:1 randomization (computer-generated, block sizes varying at random, no stratification) for each individual case. Independent assessors were blinded to group assignment. Blinded assessment of the treatment effects was ensured by videotaping the task performance and removing all hints of the treatment modality. Experimental and control interventions included the same amount of personal involvement. Neither patients nor therapists were presented with an assumption as to which intervention may be more likely to improve activities of daily living. At week 0, trial physicians completed the baseline assessment (t_0_) at the study center and patients were randomized. At weeks 1 and 2, the therapists selected two tasks that were relevant for the patient in daily life, but which he/she was no longer able to perform independently together with the patients and his or her caregiver. The baseline task performance was then videotaped. From weeks 3 to 10, patients received nine 1-hour training sessions at home. Task performance was videotaped again at week 11 (t_1_), week 16 (t_2_, primary outcome measure) and week 26 (t_3_). Therapists carried out two 1-hour refresher training sessions in weeks 19 and 20. Trial physicians completed the follow-up assessments at the study center in weeks 16 and 26 (see Table 1 for the intervention scheme).

**Table 1:** Intervention scheme

	Weeks 0–2	Weeks 3–10	Week 11	Week 16^a^	Weeks 19–20	Week 26
Measurement	t_0_	–	t_1_	t_2_	–	t_3_
Intervention	–	Nine sessions	Break		Two refresher sessions	–

^a^Primary outcome measure

### Interventions

Two separate treatment manuals for EL or TEL have been developed, pilot-tested, adapted and taught in introductory seminars. Per study site, we trained at least three therapists (occupational therapist, nurse, psychologist or social worker). To minimize contamination, we separated the main therapists for EL from the main therapists for TEL while teaching the experimental and control treatment protocols. The third therapist received both EL and TEL training, serving as a substitute; this person was not allowed to carry out more than four sessions per patient. To reduce selection bias, we assigned interventionists to EL, TEL or substitute at random.

By shared decision-making, the therapist and the patient selected two training tasks relevant for the patient’s daily living. The two selected tasks were referred to as task A and task B respectively (note that these two tasks were thus different for each patient). To do so, they used a catalog of 43 predefined tasks (20 household tasks such as doing the dishes or laying a table, 11 leisure tasks such as performing light physical exercise or taking photographs, 12 cognitively challenging tasks such as finding a bus connection or surfing the Internet). Tasks had to be independent from changing seasons or environments (e.g., not shoveling snow, not using equipment that will be renewed soon) and repeatable within 30 min (e.g., not preparing an extensive meal). Within the first three 1-hour sessions the tasks had to be selected and checked for relevance and the patient’s performance level had to show room for improvement (50–75% insufficient performance).

Each therapist’s session consisted of 1 hour for the actual training (30 min each for task A and task B) and 1 hour for documentation and travel time. If patients expressed concerns, therapists could reduce the training time to engage in motivating conversation. For the training, existing home equipment and no extra materials were used. Caregivers were not present during training.

### Errorless learning (experimental arm)

The therapist divided the task into appropriate steps, demonstrated and explained the first step, asked the patient to perform the first step and accompanied the patient’s step performance by continuous verbal instruction. As soon as the therapist anticipated a potential error, he/she intervened by giving a short demonstration of the correct performance. When the patient had performed the first step correctly, the therapist demonstrated and instructed the next step. These procedures of instruction, performance and early intervening to avoid errors were followed until the whole task was performed. The training stopped after 30 min, irrespective of how often the task or its individual steps were performed. After the fifth session, the therapist was allowed to reduce the amount of modeling and verbal instruction, but had to provide it again as soon as the patient showed potential errors, hesitated or showed uncertainty in performing the task (Table 2).

**Table 2:** Overview of the errorless learning (EL) and trial and error learning (TEL) arms of the intervention

EL intervention	TEL intervention
– Task is divided into core elements – Each step is demonstrated by the therapists accompanied with verbal instructions – The patient is then invited to perform the task step, and is verbally guided by the therapists – Only when the patient performs the first step correctly does the therapist demonstrate and instruct the next step – In case of hesitation or (near) error by the patient, the step is repeated again and the sequence is also repeated again (both with demonstrating and verbal instructions by the therapist) – From session six onward it is allowed to fade out help	– The patient must try to perform the task by himself/herself for the first 10 min, regardless of the amount of errors or hesitations – Intervention is only allowed within the first 10 min when the patient becomes irritated or frustrated – After 10 min, intervention is allowed using a stepwise approach: 1. Stimulating the patient by asking open questions 2. Summarizing what has already been done and what the task goal is 3. Giving clear verbal instruction 4. Demonstrating the task steps

### Trial and error learning (control arm)

The therapist asked the patient to perform the task and did not provide any instruction or demonstration. When the patient made an error, he/she was allowed to guess to self-correct. In the first 10-min period of the training, the therapist did not provide any support, apart from observing with interest and intervening if the patient showed signs of irritation or frustration. In the second training phase, the therapist used open-ended questions about the purpose of the task after three insufficient trials to find solutions. If the patient was still unable to perform the step, the therapist gave verbal instructions but did not demonstrate the step. This procedure of the patient performing and guessing, the therapist’s supporting open-ended questions and—if necessary—correct instructions was continued until the whole task was performed or the training stopped after 30 min (Table 2).

### Intervention adherence

Masked external raters assessed the intervention adherence by rating videos of two treatment sessions, one at the beginning and one at the end of the treatment series, for two patients of each therapist. In addition, therapists commented and self-rated their therapeutic interaction, dealing with errors and protocol adherence for each patient after a treatment series was completed in week 11. External ratings and self-ratings were scored on the same six-point scale (1 = excellent job; 6 = poor treatment).

### Outcome measures

Task performance was defined as the primary patient-related outcome measure and assessed using the CEM. All tasks of the catalog were subdivided into core elements and illustrated with detailed descriptions (see Table 3 for an example of an activity, its core elements and the individual steps). Therapists adapted this description to the individual context in the patient’s home and specified the required steps to successfully perform each core element of each chosen task. The blinded assessors used these descriptions to rate the patient’s actual performance of each core element using a seven-point scale for each task (1 = not performed at all as trained by the therapist; 7 = performed exactly as trained by the therapist) [7].

**Table 3:** Example of the ‘Making a telephone call’ activity divided into different core elements

	Core element			
	Get the number	Dial the number	Make conversation and end call	End task
Possible steps	– Take a telephone book or mobile phone – Search the telephone number in the mobile phone or phonebook – Write the number on a piece of paper	– Press the correct numbers on the telephone to dial the number OR – Select the correct name in the telephone book of the telephone – Press the correct button to make the telephone call	– Talk into the telephone OR – Talk into the telephone using the ‘speaker’ function – End the call by pressing the correct button	– Place the telephone back in the right position – Put away the telephone book and piece of paper

Secondary outcomes were daily functioning as measured with the IDDD [11], resource utilization (Resource Utilization in Dementia (RUD)) [12, 13] and satisfaction with treatment measured with a verbal rating scale (ranging from 1 = very satisfied to 5 = very unsatisfied). Furthermore, we assessed several control measures: cognitive status (MMSE [8]), dementia stage (Reisberg Clinical Dementia Rating), challenging behavior assessed by the Neuropsychiatric Inventory (NPI) [14, 15] and treatment costs using a cost unit rate of €60 per treatment hour including all costs (personnel, material, travel, overheads). In the case of group differences, these control measures can be used to adjust for potential confounding. Death, nursing home admissions and nonelective hospital admissions were defined as serious adverse events.

### Statistical analyses

A sample size of 80 participants per treatment arm was calculated for the detection of small effect sizes (*f* = 0.10) in an analysis of variance with two groups and two repeated measurements at baseline and week 16 hypothesizing α = 0.05, a power of 0.8 and a correlation of 0.6 between the measurement points (total *n* = 160). Overall efficacy of treatment was assessed by conducting multivariate analysis of covariance (ANCOVA) controlling for pretreatment scores on all outcome measures and considering all standards for the testing of assumptions. The multivariate analyses were done separately for primary and secondary outcomes because of the different numbers of measurement points. For the primary outcome, we performed an intention-to-treat (ITT) analysis on all randomized patients not dropped out at week 16. We used 10 multiple imputations with the Full Information Maximum Likelihood method when data were missing in single items or scales at week 16 or week 26 and for complete dropouts at week 26. The missing-data mechanism is ignorable if data are missing at random [16]. Missing data can be considered Missing Completely At Random (MCAR) if the probability that data are missing does not depend on observed or unobserved data. We used Little’s MCAR test to examine whether our missing pattern was completely at random. The control measures cognitive status (MMSE [8]), dementia stage (Reisberg Clinical Dementia Rating) and challenging behavior (NPI) [14, 15] were analyzed for group differences at baseline and after the intervention. We used SPSS 23.0 and a two-tailed α = 0.05 for all statistical analysis.

## Results

### Recruitment, patient flow and baseline characteristics

The recruitment period lasted 3.5 years from April 2012 to September 2015. Two initial study sites received introduction and training but could not recruit patients; one site due to lack of access to eligible patients, one site because one trained therapist decided to withdraw. Thereafter, a new study site (Mannheim) was recruited and enrolled their first patient in May 2013.

**Fig. 1:**
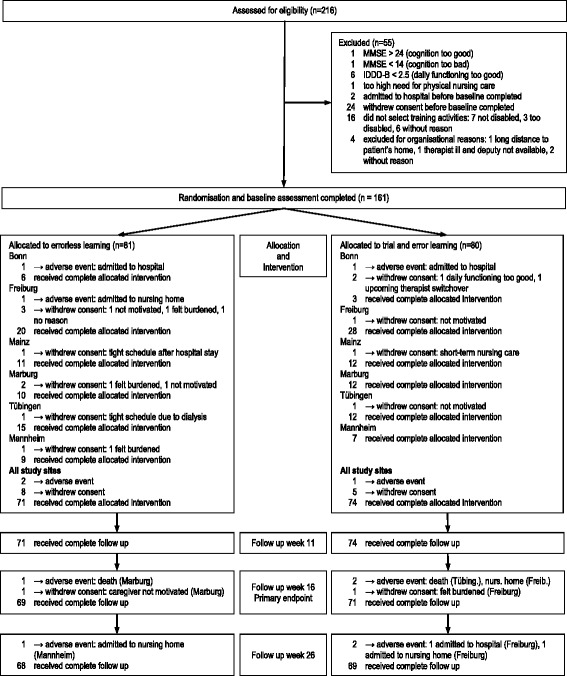
Flow of participants through the trial. *IDDD* Interview for Deterioration in Daily Living Activities, *MMSE* Mini Mental State Examination

From 161 randomized participants, 140 and 137 respectively received a follow-up assessment at week 16 and week 26 (attrition rate 13.0% and 14.9%; Fig. 1). Reasons for drop out were death (1 EL; 1 TEL), nonelective admission to hospital (1 EL; 2 TEL), admission to nursing home (2 EL; 2 TEL) and withdrawal (9 EL; 6 TEL). Group differences in the baseline characteristics of patients, caregivers and therapists were clinically not relevant (Table 4 and Additional file 1: Table S1).

**Table 4:** Demographic and clinical characteristics at baseline

	Errorless learning			Trial and error learning		
	Completers week 16 (*n* = 69)	Dropouts week 16 (*n* = 12)	Total (*n* = 81)	Completers week 16 (*n* = 71)	Dropouts week 16 (*n* = 9)	Total (*n* = 80)
Patient—demographic characteristics						
Age (years)	76.7 (8.0)	79.3 (6.1)	77.1 (7.8)	76.2 (6.5)	75.2 (9.4)	76.1 (6.8)
Sex (female)	40 (58)	6 (50)	46 (57)	40 (56)	6 (67)	46 (58)
School						
No school graduation	2 (2.9)	0 (0.0)	2 (2.5)	1 (1.4)	0 (0.0)	1 (1.3)
Middle school graduation (9 or 10 years)	60 (87.0)	11 (91.7)	71 (87.7)	52 (73.2)	7 (77.8)	59 (73.8)
High school graduation (12 or 13 years)	7 (10.1)	1 (8.3)	8 (9.9)	18 (25.4)	2 (22.2)	20 (25.0)
Vocational education						
Not completed	17 (24.6)	2 (16.7)	19 (23.5)	12 (16.9)	3 (33.3)	15 (18.8)
Completed	52 (75.4)	10 (83.3)	62 (76.5)	59 (83.1)	6 (66.7)	65 (81.3)
Patient—clinical characteristics						
TMT number of missing data	2	–	2	3	–	3
TMT number of not completed (>240 sec)	19	2	21	12	1	13
TMT number of completed (≤240 sec)	48	10	58	56	8	64
TMT completed (sec)	92.6 (40.9)	122.6 (61.6)	97.8 (45.9)	106.0 (50.8)	106.8 (45.3)	106.1 (49.8)
MMSE	19.8 (3.3)	19.1 (3.0)	19.7 (3.2)	19.7 (3.3)	20.3 (3.6)	19.8 (3.3)
Reisberg Clinical Dementia Rating	4.3 (0.6)	4.3 (0.5)	4.3 (0.7)	4.3 (0.7)	4.3 (0.7)	4.3 (0.7)
GDS	2.7 (1.7)	2.8 (1.9)	2.7 (1.9)	2.8 (2.2)	2.6 (2.1)	2.8 (2.2)
Years since dementia onset	2.2 (2.3)	1.8 (1.3)	2.1 (2.1)	1.5 (1.5)	3.3 (4.3)	1.7 (2.1)
Number of patients without additional diagnosis	27 (39.1)	4 (33.3)	31 (38.3)	30 (42.3)	4 (44.4)	34 (42.5)
Number of patients with 1–3 additional diagnoses	33 (47.8)	6 (50.0)	39 (48.1)	33 (46.5	4 (44.4)	37 46.3)
Number of patients with ≥4 additional diagnoses	9 (13.0)	2 (16.7)	11 (13.6)	8 (11.3)	1 (11.1)	9 (11.3)
Primary caregiver						
Age (years)	62.3 (13.5)	65.4 (12.9)	62.7 (13.4)	62.9 (13.8)	60.2 (12.5)	62.6 (13.6)
Sex (female)	25 (36.2)	4 (33.3)	29 (35.8)	26 (36.6)	3 (33.3)	29 (36.3)
Relation						
Spouse	35 (50.7)	7 (58.3)	42 (51.9)	41 (57.7)	4 (44.4)	45 (56.3)
(Grand) Child	30 (43.5)	4 (33.3)	34 (42.0)	27 (38.0)	5 (55.6)	32 (40.0)
Others	4 (5.8)	1 (8.3)	5 (6.2)	3 (4.2)	0 (0.0)	3 (3.8)
Living together	47 (68.1)	9 (75.0)	56 (69.1)	47 (66.2)	5 (55.6)	52 (65.0)
Caring for the patient (months)	26.4 (26.8)	33.1 (34.9)	27.4 (28.0)	21.9 (18.7)	33.4 (37.2)	23.2 (21.5)

Data presented as mean (standard deviation) or number (percentage)

*TMT* Trial Making Test, *MMSE* Mini Mental State Examination, *GDS* Geriatric Depressions Scale

### Intervention delivery and adherence

From the 81 randomized patients in the EL group, all sessions were completed in full for 71 persons. In five cases, not all sessions were completed but a sufficient number (≥9 sessions) was completed. In another five cases, an insufficient number of sessions (three, four, five, five or six sessions) had taken place. From the 80 cases randomized to TEL, the intervention was fully completed in 74 cases and sufficiently completed in two cases (≥9 sessions). Four cases received an insufficient treatment (three, four, five or six sessions). No patient changed from the EL to TEL condition or from the TEL to EL condition.

Tasks that were trained the most included following written instructions to perform light exercises (23 EL, 27 TEL), making a telephone call (15 EL, 17 TEL), selecting a specific TV broadcast (11 EL, 16 TEL), writing a shopping list (9 EL, 15 TEL), finding a telephone number (6 EL, 16 TEL), playing a DVD (11 EL, 1 TEL), playing a CD at a convenient volume (9 EL, 2 TEL) and coloring an outlined picture (5 EL, 5 TEL).

After the last treatment session, therapists rated themselves on three items (therapeutic interaction, dealing with errors and manual adherence). Each of these items was scored on a six-point scale (1 = excellent, 2 = good, 3 = adequate, 4 = sufficient, 5 = insufficient, 6 = poor). On average, therapists in both groups self-rated their intervention adherence as ‘good’ (EL: mean 1.8, SD 0.4; TEL: mean 2.0, SD 0.5). External raters used the same three items and the same six-point scale to rate the therapists’ adherence, rating them on average as ‘good to excellent’ (EL: mean 1.5, SD 0.8; TEL: mean 1.6, SD 0.8). After study completion we asked therapists for their assumptions about the superior learning technique. Six out of eight EL therapists (three missing data) and three out of 10 TEL therapists rated the technique they had performed as superior.

### Outcomes

**Fig. 2:**
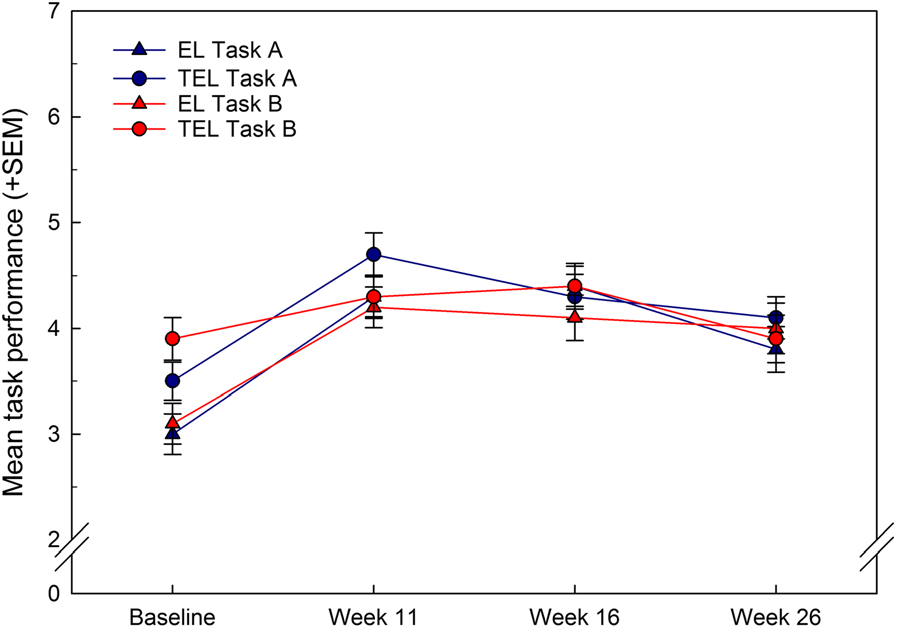
Primary outcome measure (performance on task A and task B; mean + SEM) for the errorless (*EL*) and trial and error (*TEL*) treatment arms at baseline and weeks 11, 16 and 26

For the primary outcome, ITT analysis of the 140 participants with a week 16 follow-up assessment showed significantly improved task performance of the self-selected task A and task B in both groups from baseline to week 16 (standardized effect size (95% CI): task A, 0.61 (0.37–0.85); task B, 0.47 (0.23–0.71)) and to week 26 (task A, 0.41 (0.17–0.64); task B, 0.26 (0.03–0.50)). No significant time by treatment group interaction was found and no differences were found between task A and task B (Fig. 2 and Table 5). The assumptions for multiple imputation were fulfilled as the Missing Completely At Random Test (Little’s MCAR test) showed a missing pattern completely at random (χ^2^ = 102.4, df = 102, *p* = 0.471).

**Table 5:** Patient-related outcomes following structured relearning of individually daily living tasks^a^

			Baseline		Week 11			Week 16			Week 26		
	Sample errorless	Sample trial and error	Errorless,	Baseline trial and error,	Errorless,	Trial and error,	Group ∆,	Errorless,	Trial and error,	Group ∆,	Errorless,	Trial and error,	Group ∆,
	(*N*)	(*N*)	mean (SD)	mean (SD)	mean (SD)	mean (SD)	mean (95% CI)	mean (SD)	mean (SD)	mean (95% CI)	mean (SD)	mean (SD)	mean (95% CI)
Primary patient-related outcome													
Task performance rating video A (1 = worst, 7 = best)	69	71	3.0 (1.6)	3.5 (1.5)	4.3 (1.7)	4.2 (1.7)	–0.1 (–0.6, 0.5)	4.4 (1.8)	4.1 (1.8)	0.3 (–0.3, 0.9)	3.8 (1.8)	4.0 (1.7)	0.3 (–0.5, 0.8)
Task performance rating video B (1 = worst, 7 = best)	69	71	3.1 (1.6)	3.9 (1.7)	4.7 (1.6)	4.3 (1.6)	–0.4 (–0.9, 0.1)	4.3 (1.8)	4.4 (1.6)	–0.1 (–0.5, 0.7)	4.1 (2.0)	3.9 (1.9)	–0.2 (–0.9, 0.5)
Secondary patient-related outcomes													
IDDD A initiative (0 = worst, 36 = best)	69	71	18.4 (6.6)	18.2 (5.8)				17.7 (7.0)	16.8 (7.2)	–0.9 (–3.3, 1.5)	16.2 (7.5)	15.7 (7.4)	–0.4 (–3.1, 2.0)
IDDD B performance (44 = worst, 0 = best)	69	71	21.3 (6.0)	21.4 (7.2)				21.0 (8.6)	20.9 (9.0)	0.0 (–3.0, 2.9)	22.3 (10.2)	23.6 (10.1)	1.4 (–2.1, 4.8)
Treatment satisfaction (5 = worst, 1 = best)	69	71			1.5 (0.6)	1.5 (0.5)	0.0 (–0.2, 0.2)				1.5 (0.9)	1.4 (0.7)	–0.1 (–0.4, 0.2)
Control measures													
MMSE (0 = worst, 30 = best)	69	71	19.8 (3.3)	19.7 (3.3)				19.0 (4.8)	19.6 (4.2)	0.6 (–0.9, 2.1)	18.2 (5.2)	18.9 (5.1)	0.7 (–1.1, 2.4)
Reisberg Clinical Dementia Rating (1 = worst, 7 = best)	69	71	4.3 (0.6)	4.3 (0.7)				4.4 (0.6)	4.4 (0.7)	0.1 (–0.2, 0.3)	4.4 (0.6)	4.4 (1.0)	0.0 (–0.3, 0.3)
NPIQ (36 = worst, 0 = best)	69	71	7.2 (4.0)	7.5 (5.2)				7.9 (5.2)	8.1 (5.2)	–0.1 (–1.7, 1.8)	8.0 (5.4)	8.6 (6.5)	0.6 (–1.5, 2.7)

*SD* standard deviation, *IDDD* Interview for Deterioration in Daily Living Activities, *MMSE* Mini Mental State Examination, *NPIQ* Neuropsychiatric Inventory Questionnaire

^a^Intention-to-treat analysis of 140 participants with follow-up data at week 16 and multiple imputations when data were missing in single measurement instruments

For secondary outcome and control measures, the patient’s need for assistance (measured with the IDDD), cognition (measured with the MMSE), challenging behavior (measured with the NPIQ) and Satisfaction with Treatment verbal rating scale, as well as treatment costs and resource utilization (measured with the RUD), remained stable over 26 weeks and did not significantly differ by treatment group or measurement time point (Table 5). Patients of both groups rated satisfaction with treatment as very good. Costs were similar for EL and for TEL (€1907 and €1897, respectively) (see Additional file 2: Table S2). Because we found no group differences at baseline or after the intervention on any of the control measures (Table 5), we did not use them for adjusting the primary outcome for confounding.

Study sites reported four serious adverse events in the EL group (one death, one nonelective hospital admission, two nursing home admissions) and five in the TEL group (one death, two nonelective hospital admissions, two nursing home admissions). Study site leaders judged all serious adverse events as unrelated to the study treatment or assessment.

## Discussion

This is the first large RCT on EL as a method to teach persons with dementia activities of daily living in their own environment. The objective of this multicenter REDALI-DEM trial was to evaluate whether EL or TEL demonstrates superior effects on the performance of two relevant activities of daily living in persons with mild to moderate AD living at home. Results showed an improved post-treatment performance of daily living tasks in both arms, but EL was not found to be superior to TEL. The relearning of activities did not affect the patients’ initiative or need for assistance in activities of daily living. Both EL and TEL were very well accepted by the patients and the costs did not differ between both treatments. Although serious adverse events occurred, these were judged unrelated to the intervention. The fact that improved task performance in both treatment arms did not lead to improvement on secondary outcomes indicates that no generalization effects on daily life functioning were found, but only improvements on the trained tasks. Note that the lack of to be expected generalization effects has been put forward as a limitation of EL previously [4].

These results are not in agreement with most earlier findings on the effects of EL, because previous reviews on EL suggested superior results for EL compared with TEL. However, most previous studies were small-scale trials or proof-of-principle studies in which patients were taught artificial tasks that had marginal relevance to them (such as learning an artificial word list). Our hypothesis that EL would be a more effective teaching method for persons with dementia was based on these earlier findings. However, this hypothesis is not confirmed in this first adequately powered, rigorously designed and well-performed multicenter RCT.

Recent studies that have used procedural tasks or skills to examine the effects of EL in patients with dementia showed mixed results [5, 17]. One explanation for these mixed findings may lie in the nature of the tasks. That is, there is some evidence that EL works through the facilitation of implicit, automatic learning processes, which have been shown to be intact in patients with dementia [18]. Possibly, the procedural nature of the tasks that were trained in the current RCT may in itself have already facilitated learning, irrespective of the error reduction aspect. Indeed, learning has taken place in both treatment arms, which is in line with this view. Moreover, both treatment procedures could be categorized as forms of ‘structured learning’. That is, therapists adopted a step-by-step approach, provided feedback and stimulated engagement in the task. This structuring may have optimized learning in itself, resulting in better post-treatment task performance, an effect that was also maintained at the follow-up assessment. In addition, to measure our primary outcome a recently developed rating scale was used: the Core Elements Method (CEM). The pilot phase was therefore also used to validate this newly developed outcome measure. The interrater reliability and concurrent validity of the CEM and TPS were analyzed and compared [7]. Based on these results, the TPS and CEM were found to be equally valid for the assessment of task performance in people with dementia. However, the CEM was found to be less complex and less time-consuming compared with the TPS, and therefore used in the current RCT. Although result from this previous pilot phase showed excellent validity and reliability, the current results have to be interpreted with some caution because its psychometric properties have only been examined in one study.

Strengths of the current study include the naturalistic setting of the intervention. That is, the intervention was carried out in the patients’ own homes, using tasks that were relevant for them to acquire. Patients also appreciated the intervention very much. This may have also promoted learning in both arms, obscuring a potential superior effect of error reduction. In addition, the large sample size and the low drop-out rate can be considered strengths of the study. The low drop-out rate prevented attrition bias and justifies that we did not include all randomized patients in our ITT analysis, but only those with data for at least at two time points (baseline and primary outcome time point at week 16). Data imputation for participants with data for only one time point is prone to adverse events (three patients). Note that all other dropouts withdrew their consent.

All persons involved were blinded to our hypotheses and the raters who assessed the primary outcome were also fully blinded to treatment arms and the hypothesis. Treatment adherence of the therapists was monitored using self-ratings and external ratings, showing good treatment adherence. The planned sample size was reached in this RCT and the findings are reported according to the CONSORT guidelines [19], including long-term results, treatment costs and adverse events.

Limitations include the heterogeneity of the tasks that were trained. Tasks like making a grocery list or planning a trip may have had more degrees of freedom than straightforward ‘stimulus-response’ tasks such as dialing a telephone number or playing a DVD. A methodological limitation was that patients and therapists by definition were not blinded to the treatment itself, although measures were taken to prevent cross-over effects by letting therapists give only one type of intervention.

## Conclusion and future research

Persons with dementia can still be retrained in performing activities of daily living using structured learning, with effects being maintained for 6 months. However, EL had no additional effect over TEL. Future research should examine whether the effectiveness of structured learning depends on patient-specific or task-specific characteristics.

## Additional files

Additional file 1: Table S1. Presenting characteristics of the therapists participating in both learning conditions (years in the field, qualification, cases trained and sex). (DOCX 13 kb)

Additional file 2: Table S2. Presenting an overview of resource utilization (treatment hours and costs, intensity of professional and primary care and use of dementia related medicines) following structured relearning of individually selected daily living tasks for both learning conditions at baseline and weeks 11, 16 and 26. (DOCX 16 kb)

## Abbreviations

AD: Alzheimer’s dementia
CI: Confidence interval
EL: Errorless learning
GDS: Geriatric Depression Scale
IDDD: Interview for Deterioration in Daily Living Activities
ITT: Intention to treat
MMSE: Mini Mental State Examination
RCT: Randomized controlled trial
REDALI-DEM: RElearning methods on DAily LIving task performance of persons with DEMentia
TEL: Trial and error learning
