# Supplementary material for: Structured relearning of activities of daily living in dementia: the randomized controlled REDALI-DEM trial on errorless learning
Source: Alzheimers Res Ther. 2017 Mar 23;9:22. doi: 10.1186/s13195-017-0247-9 (PMC5364615; doi:10.1186/s13195-017-0247-9)
Supplement: Supplementary file 1 — Presenting characteristics of the therapists participating in both learning conditions (years in the field, qualification, cases trained and sex). (DOCX 13 kb) [file 13195_2017_247_MOESM1_ESM.docx]

**Additional file 1: Table S1** Characteristics of the therapists

| **Site** | **Male/ Female*** | **Qualification** | **Years in the field** | **Cases** |
| --- | --- | --- | --- | --- |
| **Errorless learning** | | | | |
| Bonn | F | OT | 5 | 7 |
| Freiburg | F | OT | 10 | 1 |
| Freiburg | F | OT | 6 | 21 |
| Freiburg | F | OT | 3 | 2 |
| Mainz | M | OT | 9 | 11 |
| Mainz | M | PS | 1 | 1 |
| Marburg | F | PS | 1 | 9 |
| Marburg | M | PS | 1 | 3 |
| Tübingen | F | OT | 18 | 12 |
| Tübingen | F | OT | 4 | 4 |
| Mannheim | M | OT | 3 | 10 |
| Total Mean (SD) |  |  | 5.5 (5.1) | 7.4 (6.1) |
| **Trial and error learning** | | | | |
| Bonn | F | OT | 6 | 2 |
| Bonn | F | OT | 3 | 4 |
| Freiburg | F | OT | 6 | 29 |
| Mainz | F | PS | 1 | 5 |
| Mainz | F | PS | 3 | 3 |
| Mainz | F | PS | 1 | 5 |
| Marburg | F | OT | 15 | 12 |
| Tübingen | M | PS | 4 | 6 |
| Tübingen | F | SO | 5 | 7 |
| Mannheim | F | OT | 4 | 7 |
| Total Mean (SD) |  |  | 4.8 (4.0) | 8.0 (7.9) |

*M: Male; F: Female; Qualification OT: Occupational Therapist; PS: Psychologist; SO: Social Worker
