# Supplementary material for: Structured relearning of activities of daily living in dementia: the randomized controlled REDALI-DEM trial on errorless learning
Source: Alzheimers Res Ther. 2017 Mar 23;9:22. doi: 10.1186/s13195-017-0247-9 (PMC5364615; doi:10.1186/s13195-017-0247-9)
Supplement: Supplementary file 2 — Presenting an overview of resource utilization (treatment hours and costs, intensity of professional and primary care and use of dementia related medicines) following structured relearning of individually selected daily living tasks for both learning conditions at baseline and weeks 11, 16 and 26. (DOCX 16 kb) [file 13195_2017_247_MOESM2_ESM.docx]

**Additional file 2: Table S2:** Resource utilization following structured relearning of individually selected daily living tasks

|  | Sample | Sample | Baseline | Baseline | Week 11 | Week 11 | Week 11 | Week 16 | Week 16 | Week 16 | Week 26 | Week 26 | Week 26 |
| --- | --- | --- | --- | --- | --- | --- | --- | --- | --- | --- | --- | --- | --- |
|  | errorless | trial & error | errorless | trial & error | errorless | trial & error | group ∆ | errorless | trial & error | group ∆ | errorless | trial & error | group ∆ |
|  | N | N | mean (SD) | mean (SD) | mean (SD) | mean (SD) | mean [95%-CI] | mean (SD) | mean (SD) | mean [95%-CI] | mean (SD) | mean (SD) | mean [95%-CI] |
| **Resource Utilization** |  |  |  |  |  |  |  |  |  |  |  |  |  |
| Treatment hours (SD) | 69 | 71 |  |  | 25.9 (0.8) | 26.0 (0.6)) | 0.1 [-0.1,0.4] |  |  |  | 5.9 (0.3) | 5.6 (1.2) | -0.3 [-0.6, 0.0] |
| Treatment costs, EURO (SD) | 69 | 71 |  |  | 1551.30 (47.5) | 1560.0 (35.1)) | 8.7 [-5.3,22.7] |  |  |  | 355.7 (18.7) | 337.2 (71.3) | -18.5 [-35.9,-1.1] |
| RUD resource utilization during last two weeks |  |  |  |  |  |  |  |  |  |  |  |  |  |
| Nights in hospital (SD) | 69 | 71 | 0.3 (1.7) | 0.4 (2.3) |  |  |  | 0.0 (0.0) | 0.13 (1.1) | 0.1 [-0.1, 0.4] | 0.1 (0.7) | 0.2 (1.6) | 0.1 [-0.3;0.5] |
| Nights in short-term nursing care (SD) | 69 | 71 | 0.0 (0.0) | 0.0 (0.0) |  |  |  | 0.0 (0.0) | 0.0 (0.0) | 0.0 [] | 0.2 (1.7) | 0.2 (1.3) | -0.0 [-0.6;0.5] |
| Contacts with general practitioner (SD) | 69 | 71 | 0.5 (0.6) | 0.4 (0.6) |  |  |  | 0.5 (0.8) | 0.4 (0.6) | -0.1 [-0.3, 0.1] | 0.5 (0.7) | ß.4 (0.5) | -0.1 [-0.3;0.1] |
| Contacts with specialist (SD) | 69 | 71 | 0.4 (0.7) | (0.4 (1.0) |  |  |  | 0.1 (0.3) | 0.3 (0.5) | 0.2 [0.0, 0.4] | 0.2 (0.4) | 0.2 (0.5) | 0.0 [-0.2,0.1] |
| Number of purchased devices (SD) | 69 | 71 | 0.1 (0.4) | 0.1 (0.3) |  |  |  | 0.1 (0.4) | 0.1 (0.3) | 0.0 [-0.1,0.1] | 0.1 (0.4) | 0.1 (0.4) | 0.0 [-0.1,0.1] |
| Hours community therapy service (SD) | 69 | 71 | 0.3 (0.9) | 0.2 (1.0) |  |  |  | 0.2 (0.5) | 0.2 (1.0) | 0.0 [-0.2,0.3] | 0.2 (0.6) | 0.2 (0.7) | 0.0 [-0.2,0.2] |
| Hours community group service (SD) | 69 | 71 | 0.6 (2.0) | 2.4 (4) |  |  |  | 1.6 (5.6) | 3.8 (10.2) | 2.3 [-0.5,5.0] | 2.4 (7.1) | 4.4 (11.1) | 2.0 [-1.1,5.1] |
| Hours community nursing service (SD) | 69 | 71 | 0.7 (2.3) | 0.6 (2.3) |  |  |  | 0.8 (2.4) | 0.9 (2.1) | 0.0 [-0.7,0.8] | 1.1 (3.4) | 0.8 (2.0) | -0.3 [-1.2,0.7] |
| Hours community support service (SD) | 69 | 71 | 7.3 (39.8) | 3.6 (11.7) |  |  |  | 7.8 (40.0) | (11.2 (53.6) | 3.4 [-12.4,19.2] | 7.9 (41.7) | 12.9 (55.1) | 5.0 [-11.3,21.4] |
| Hours of active support by primary caregiver (SD) | 68/67/66* | 71/69/67* | 5.7 (4.4) | 6.4 (4.8) |  |  |  | 7.5 (5.7) | 8.2 (5.4) | 0.7 [-1.1, 2.6] | 8.4 (5.2) | 8.4 (5.2) | 0.0 [-1.7,1.8] |
| Hours of supervision by primary caregiver (SD) | 68/67/66* | 71/69/67* | 0.7 (4.1) | 0.3 (2.8) |  |  |  | 5.5 (6.0) | 4.1 (5.5) | -1.4 [-3.3, 0.6] | 5.0 (5.2) | 4.4 (5.1) | -0.7 [-2.4,1.1] |
|  |  |  |  |  |  |  |  |  |  |  |  |  |  |
| Intake of cholinergic drugs, mg per day (SD)  Donezepil  Rivastigmin  Galantamin  Memantin, Ebixa  Memantin, Axura | 69/67/66* | 71/69/68* | 3.0 (4.2)  0.6 (2.2)  0.7 (4.1)  0.1 (0.8)  3.0 (6.8) | 3.3 (4.3)  0.9 (2.6)  0.3 (2.8)  0.6 (3.3)  1.7 (5.6) |  |  |  | 4.6 (5.0)  0.6 (2.3)  0.7 (4.1)  0.5 (2.7)  3.5 (8.4) | 4.9 (4.8)  1.1 (3.1)  0.4 (2.9)  0.3 (2.4)  2.3 (6.5) | 0.2 [-1.4,1.9]  0.3 [-0.4,1.4]  -0.4 [-1.6,0.8]  -0.2 [-1.0,0.7]  -1.2 [-3.7,1.4] | 4.8 (4.8)  0.5 (2.1)  0.4 (2.9)  1.1 (3.6)  3.1 (7.4) | 5.0 (4.7)  1.2 (3.1)  0.7 (4.1)  0.3 (2.4)  2.1 (6.1) | 0.2 [-1.4,1.8]  0.7 [-0.2,1.6]  -4 [-1.6,0.8]  -0.8 [-1.8,0.3]  -1.0 [-3.4,1.3] |

*numbers represent N at: baseline / week 16 / week 26. Abbreviations: SD, Standard Deviation; *= P<0.05
